# Monocyte subset distribution in patients with stable atherosclerosis and elevated levels of lipoprotein(a)

**DOI:** 10.1016/j.jacl.2015.04.005

**Published:** 2015

**Authors:** Konstantin A. Krychtiuk, Stefan P. Kastl, Sebastian L. Hofbauer, Anna Wonnerth, Georg Goliasch, Maria Ozsvar-Kozma, Katharina M. Katsaros, Gerald Maurer, Kurt Huber, Elisabeth Dostal, Christoph J. Binder, Stefan Pfaffenberger, Stanislav Oravec, Johann Wojta, Walter S. Speidl

**Affiliations:** a Division of Cardiology, Department of Internal Medicine II, Medical University of Vienna, Vienna, Austria; b Ludwig Boltzmann Cluster for Cardiovascular Research, Vienna, Austria; c Department of Laboratory Medicine, Medical University of Vienna, Vienna, Austria; d 3rd Medical Department, Wilhelminenhospital, Vienna, Austria; e Krankenanstalten Dr. Dostal, Vienna, Austria; f 2nd Department of Internal Medicine, Faculty of Medicine, Comenius University, Bratislava, Slovakia; g Core Facilities, Medical University of Vienna, Vienna, Austria

**Keywords:** Lipoprotein(a), Atherosclerosis, Coronary artery disease, Monocyte subsets, Oxidized phospholipids

## Abstract

**Background:**

Lipoprotein(a) (Lp(a)) is a proatherogenic plasma lipoprotein currently established as an independent risk factor for the development of atherosclerotic disease and as a predictor for acute thrombotic complications. In addition, Lp(a) is the major carrier of proinflammatory oxidized phospholipids (OxPL). Today, atherosclerosis is considered to be an inflammatory disease of the vessel wall in which monocytes and monocyte-derived macrophages are crucially involved. Circulating monocytes can be divided according to their surface expression pattern of CD14 and CD16 into at least 3 subsets with distinct inflammatory and atherogenic potential.

**Objective:**

The aim of this study was to examine whether elevated levels of Lp(a) and OxPL on apolipoprotein B-100–containing lipoproteins (OxPL/apoB) are associated with changes in monocyte subset distribution.

**Methods:**

We included 90 patients with stable coronary artery disease. Lp(a) and OxPL/apoB were measured, and monocyte subsets were identified as classical monocytes (CMs; CD14++CD16−), intermediate monocytes (IMs; CD14++CD16+), and nonclassical monocytes (NCMs; CD14+CD16++) by flow cytometry.

**Results:**

In patients with elevated levels of Lp(a) (>50 mg/dL), monocyte subset distribution was skewed toward an increase in the proportion of IM (7.0 ± 3.8% vs 5.2 ± 3.0%; *P* = .026), whereas CM (82.6 ± 6.5% vs 82.0 ± 6.8%; *P* = .73) and NCM (10.5 ± 5.3 vs 12.8 ± 6.0; *P* = .10) were not significantly different. This association was independent of clinical risk factors, choice of statin treatment regime, and inflammatory markers. In addition, OxPL/apoB was higher in patients with elevated Lp(a) and correlated with IM but not CM and NCM.

**Conclusions:**

In conclusion, we provide a potential link between elevated levels of Lp(a) and a proatherogenic distribution of monocyte subtypes in patients with stable atherosclerotic disease.

## Highlights

• Lipoprotein(a) serves as an independent risk factor in atherosclerotic disease.
• Monocyte subsets exhibit distinct inflammatory and atherogenic properties.
• Patients with elevated levels of Lp(a) show a shift towards intermediate monocytes.
• This association was independent of clinical properties and inflammatory markers.
• Those patients also exhibited higher OxPL/apoB concentrations.

## Introduction

Despite major advances in vascular medicine including high-dose statin treatment, cardiovascular diseases remain the leading cause of death in the Western world.^1^ Lipoprotein(a) (Lp(a)) is a plasma lipoprotein that consists of a cholesterol-rich, low-density lipoprotein (LDL)-like particle and an additional protein apolipoprotein(a), which is covalently bound to the ApoB of the LDL-like particle.^2,3^ Today it is established as an independent risk factor for the development of stable ischemic disease and myocardial infarction.^2–4^ Genetic studies suggest causality; however, mechanistic insights are scarce.^5,6^ The European Atherosclerosis Society suggests to measure Lp(a) in all intermediate or high-risk patients and advises a desirable level of less than 50 mg/dL (<80th percentile).^2^

Monocytes are crucially involved in practically all stages of atherogenesis as the cellular drivers of vascular inflammation hallmarking atherosclerotic disease.^7,8^ Monocytes represent a heterogeneous cell population, reflected by their differential surface expression pattern of CD14 and CD16.^9^ CD16-positive monocytes account for more than 10% of all monocytes and were soon suggested to be proinflammatory cells characterized by a pronounced inflammatory answer on stimulation.^10,11^ Furthermore, the proportion of these cells was expanded in various inflammatory diseases, including a mixed patient cohort with coronary artery disease (CAD).^12^ Accumulating evidence suggested a further division of CD16-positive cells into 2 subtypes of cells, which was finally officially acknowledged in a consensus article under the auspices of the Nomenclature Committee of the International Union of Immunological Societies proposing the following classification: CD14++CD16− (classical monocytes [CMs]), CD14++CD16+ (intermediate monocytes [IMs]), and CD14+CD16++ (nonclassical monocytes [NCMs]).^13^ CD16-positive monocytes were shown to correlate with body mass index and intima media thickness in apparently healthy adults and were associated with plaque phenotype in patients with both, stable and unstable, atherosclerotic disease.^14–16^ NCMs correlated with total cholesterol (TC) and triglycerides, whereas a negative association with high-density lipoprotein (HDL) levels was shown.^17,18^ The largest study evaluating monocyte subset distribution as a biomarker for cardiovascular disease prediction in more than 900 stable CAD patients showed that an increased proportion of IM was associated with increased cardiovascular events.^19^

Lp(a) is the major carrier of oxidized phospholipids (OxPL) that have been shown to act proinflammatory and to activate monocytes.^20^ Insights into the mechanism shifting monocyte subset distribution toward a more proinflammatory distribution are limited. The aim of this study was therefore to investigate whether patients with stable CAD at high cardiovascular risk evidenced by elevated Lp(a) levels show a shift in monocyte subset distribution toward a proatherogenic profile and whether monocyte subset distribution is associated with OxPL on apolipoprotein B-100–containing lipoproteins (OxPL/apoB).

## Methods

### Subjects and study design

Between September 2009 and April 2010, we included all consecutive patients with stable CAD admitted to the General Hospital of Vienna for elective coronary angiography. The study complies with the Declaration of Helsinki and was approved by the ethical committee of the Medical University of Vienna. All patients, female and male, aged older than 18 y with stable CAD who gave written consent were included in this study. To avoid any impact of recent acute inflammatory conditions, we excluded patients who experienced a recent acute coronary syndrome, defined as ST-elevating myocardial infarction, non–ST-elevating myocardial infarction, or unstable angina with or without undergoing percutaneous coronary intervention within the last 3 mo. Further exclusion criteria consisted of heart failure, valvular disease, malignant disease, liver, kidney or other acute or chronic inflammatory diseases.

Arterial hypertension was defined as systolic blood pressure ≥140 mm Hg and diastolic blood pressure ≥90 mm Hg in at least 2 measurements or the current use of antihypertensive drugs. Patients were considered to be diabetic if treated for insulin or noninsulin-dependent diabetes mellitus or having a plasma fasting glucose ≥126 mg/dL in at least 2 measurements or having a glycated hemoglobin fraction of ≥6.5%. Statin treatment was noted, and treatment with atorvastatin with a dosage of at least 40 mg or rosuvastatin at a dosage of at least 10 mg daily was defined as moderate-to-high dose statin treatment. CAD extent is given as the number of epicardial coronary arteries with a ≥70% stenosis.

### Blood sampling

To exclude effects of angioplasty on monocyte subset distribution and any diurnal variation, blood was drawn in the morning before coronary angiogram. A 21-gauge butterfly needle (0.8 mm × 19 mm; Greiner Bio-One, Kremsmünster, Austria) was used for venipuncture from an antecubital vein. The initial 3 mL of blood were discarded, and blood was drawn into an EDTA tube (Greiner Bio-One) for immediate analysis by flow cytometry. An additional 3.8% sodium citrate Vacuette tube, a serum separator tube and an EDTA tube (all Greiner Bio-One) were collected, centrifuged at 3000 rpm at 4°C for 15 min and stored in aliquots at -80°C for later analysis.

### Flow cytometry

**Figure 1:**
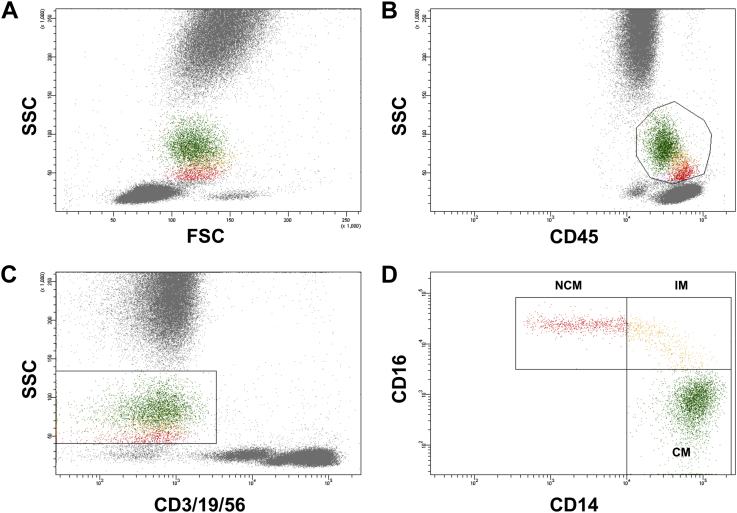
Gating strategy used for monocyte subset discrimination. Monocytes were defined as CD45 positive cells (B) exhibiting a typical forward (FSC) and sideward scatter (SSC) profile (A). To exclude possible contamination with T-cells, B-cells, and natural killer cells, cells that stained for CD3, CD19, and CD56 were excluded, respectively (C). Remaining CD45+CD3/19/56– cells with a typical FSC/SSC profile were considered monocytes and distinguished according to their CD14 and CD16 surface expression into classical monocytes (CMs; CD14++CD16−), intermediate monocytes (IMs; CD14++CD16+), and nonclassical monocytes (NCMs; CD14+CD16++) (D).

Whole blood flow cytometry for the determination of leukocyte and monocyte subset distribution was performed using a FACS Canto II with the FACS Diva Software (both Becton Dickinson). The staining and gating strategy is outlined in Figure 1 and was described previously.^21^ Briefly, 100 μL of EDTA-anticoagulated whole blood was stained with saturating concentrations of the following fluorochrome-conjugated monoclonal antibodies (mAbs): peridinin chlorophyll protein–labeled mAb for CD45 (Beckton Dickinson, catalog number 345809), fluorescein isothiocyanate–labeled mAb for CD14 (Beckton Dickinson, catalog number 345784), allophycocyanin-H7–labeled mAb for CD16 (Beckton Dickinson, catalog number 560195), allophycocyanin-labeled mAb for CD3 (Beckton Dickinson, catalog number 345767), CD19 (Beckton Dickinson, catalog number 345791), and CD56 (Beckton Dickinson, catalog number 341027) and corresponding isotype controls. After incubation for 15 min in the dark, 1.5 mL lysing solution (BD FACS lysing solution BD Biosciences) was added. After an additional 15 min of incubation in darkness, cells were washed 3 times by adding 1 mL phosphate-buffered saline and centrifugation at 820 rpm for 5 min each. Cells were then resuspended in 1 mL fixative solution (FACS Flow, reagent-grade water and BD Cellfix) for FACS-analysis. Monocytes were identified as CD45-positive and CD3-, CD19-,and CD56-negative cells exhibiting a specific forward and sideward scatter profile. Individual monocyte subsets were defined according to a recently published international consensus document as “classical monocytes” (CM; CD14++CD16−), “intermediate monocytes” (IM; CD14++CD16+), and “nonclassical monocytes” (NCM; CD14+CD16++). Absolute numbers of monocytes were calculated using leukocyte count as determined by the central laboratory and counts of CD45+ cells as determined by flow cytometry. The coefficient of variation for relative monocyte count was 4.9%.

### Lipid measurements

For all lipid measurements, only previously unthawed serum samples were used. Levels of TC (coefficient of variation [CV], 1.4%^22^), HDL cholesterol (C.V., between 5% and 6%^23^), and triglycerides(C.V., 1.3%) were measured by the general laboratory of Krankenanstalten Dr. Dostal using enzymatic methods. LDL cholesterol was calculated using the Friedewald formula when triglycerides were <400 mg/dL and measured directly when triglycerides were ≥400 mg/dL (C.V., 1.7%^24^). Lp(a) was measured by an isoform-insensitive immunonephelometric assay (Roche Diagnostics, Germany; intra-assay C.V. ranging from 0.9% to 6.2%) that measures apo(a) protein mass.

### Measurement of OxPL on apolipoprotein B-100–containing lipoproteins

OxPL/apoB was measured in EDTA plasma using the murine mAb antibody E06 that recognizes the phosphocholine group on oxidized but not on native phospholipids. A 1:100 dilution of plasma in phosphate-buffered saline is added to microtiter wells coated with mAb MB47, which binds a saturating amount of apoB-100 to each well. Finally, biotinylated E06 is used to determine OxPL/apoB in relative light units (RLUs).^25^ The intra-assay coefficient of variation was shown to range from 6% to 10%.^26^

### Measurements of cytokines and standard laboratory markers

Interleukin-6 (IL-6) was measured using a specific enzyme-linked immunosorbent assay (ELISA; Human IL-6 Quantikine high-sensitivity ELISA Kit, R&D Systems, Minneapolis, MN) with a lower limit of detection of 0.016 pg/mL. The intra-assay coefficient of variation ranges from 6.9% to 7.4%, and the inter-assay coefficient of variation ranges from 6.5% to 9.6%. Plasma levels of IL-10 were quantified using a customized multiplex assay (Luminex Assay, R&D Systems) with a lower limit of detection 0.07 pg/mL. The intra-assay coefficient of variation ranges from 3.8% to 7.3%, and the inter-assay coefficient of variation ranges from 5.1% to 12.5%. For the determination of standard laboratory markers, including high-sensitive C-reactive protein (CRP; C.V., 2.7%^27^), blood was analyzed in the central laboratory of the General Hospital of Vienna.

### Statistical analysis

Categorical variables are expressed as counts or percentages and were compared by the χ^2^ or by the Fisher exact test where appropriate. Continuous variables are given as mean ± standard deviation or median (interquartile range [IQR]). Parametric data were compared using the unpaired Student *t* test, whereas skewed data (assessed by the Kolmogorov–Smirnov test) were compared by the Mann–Whitney *U* test. Correlations were calculated using Pearson's correlation coefficient after log-transformation of skewed data. Multivariate linear regression models were calculated for circulating IMs. Clinical characteristics or lipid parameters were added to the model when they were associated with IM by a *P* value < .2. In addition, we added age and statin treatment. We also calculated stepwise multivariate linear regression models including Lp(a) and the inflammatory markers CRP, IL-6, and IL-10, respectively. A value of *P* < .05 (2-tailed) was considered statistically significant. All statistical analyses were performed with the Predictive Analysis SoftWare PASW Statistics 18.0 (IBM, Armonk, NY).

## Results

### Patient characteristics

The clinical characteristics of the population studied are given in Table 1. Patients were aged 64.1 ± 9.0 y, 80% were male, 89% had hypertension, 30% were diagnosed for having diabetes mellitus, and 23% were current smokers. All patients had angiographically proven CAD, 28% had single-vessel disease, 40% demonstrated 2 diseased vessels, and 32% had 3-vessel disease. Concerning lipid-lowering treatment, 31% of patients received moderate-to-high dose statin, 52% low-dose statin, whereas 17% of patients received no statin treatment. According to current guidelines,^2^ 21 patients (23%) had elevated levels of Lp(a) (>50 mg/dL), whereas 69 patients (77%) had normal levels of Lp(a) (≤50 mg/dL). Patients with increased Lp(a) levels showed no differences in CAD severity (*P* = .34); however, they showed a definite trend to be younger compared with patients with normal Lp(a) levels (61.0 ± 9.7 vs 65.1 ± 9.1 y; *P* = .08). Furthermore, there were no differences in demographics, risk factors, or lipid levels (Table 1).

### Association of Lp(a) serum levels and monocyte subsets

**Figure 2:**
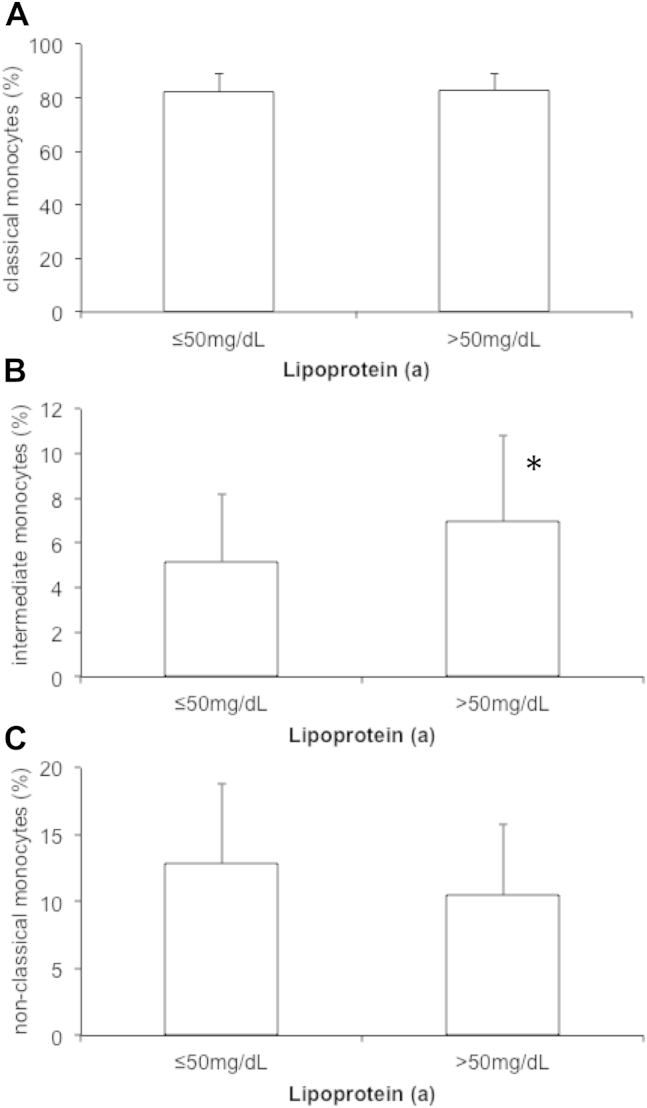
Monocyte subset distribution in patients with elevated lipoprotein(a). The distribution of classical monocytes (A), intermediate monocytes (B), and non-classical monocytes (C) in patients with normal (≤50 mg/dL) and elevated lipoprotein(a) (>50 mg/dL) were determined as described under Methods. Bar graphs indicate mean % of total monocytes and error bars represent standard deviation. *P < .05.

Monocyte subset distribution was determined by flow cytometry (Fig. 1). Mean number of CMs was 270.5 ± 142.7 cells/μL (82.1 ± 6.7% of total monocytes), mean number of circulating NCMs was 39.7 ± 28.9 cells/μL (12.3 ± 5.9% of total monocytes), and mean number of IMs was 18.7 ± 15.1 cells/μL (5.6 ± 3.3% of total monocytes). Patients with elevated Lp(a) showed a significantly higher proportion of circulating IM as compared with patients with normal Lp(a) levels (7.0 ± 3.8% vs 5.2 ± 3.0%; *P* = .026). In contrast, distribution of CMs (82.6 ± 6.5% vs 82.0 ± 6.8%; *P* = .73) and NCMs (10.5 ± 5.3 vs 12.8 ± 6.0; *P* = .10) was similar in patients with Lp(a) >50 mg/dL and Lp(a) ≤50 mg/dL (Fig. 2). Linear regression analysis revealed that the association between elevated Lp(a) and IM was independent of other lipid parameters, risk factors, and statin treatment regime (Table 2).

### OxPL on apolipoprotein B-100–containing lipoproteins is associated with IMs

**Figure 3:**
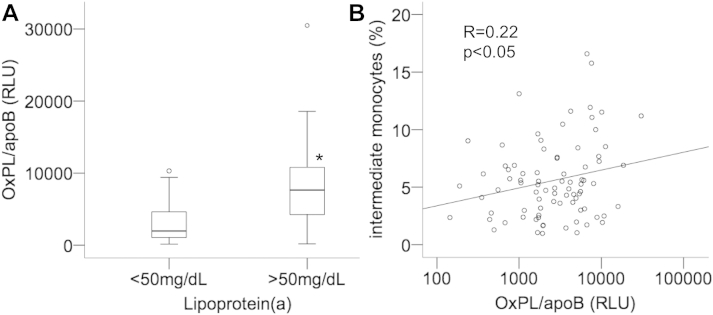
Association of oxidized phospholipids with lipoprotein(a) levels and intermediate monocytes (IMs). Plasma levels of OxPLP were determined as described under Methods in patients with normal (≤50 mg/dL) and elevated lipoprotein(a) (>50 mg/dL) (A). Correlation of oxidized phospholipid (OxPLP) with IM (B). *P < .0001.

OxPL/apoB correlated with Lp(a) (*R* = 0.43; *P* < .0001), and patients with increased Lp(a) showed significantly higher plasma levels of OxPL/apoB (7647; IQR, 4192–10915 RLU) as compared with patients with normal Lp(a) levels (1966; IQR, 1051–4772 RLU; *P* < .0001; Fig. 3A). Interestingly, OxPL/apoB showed a significant correlation with IM (*R* = 0.22; *P* < .05; Fig. 3B) but not with CM (R = −0.17; *P* = .13) and NCM (R = 0.07; *P* = .53).

### Association of circulating inflammatory markers and Lp(a)

**Figure 4:**
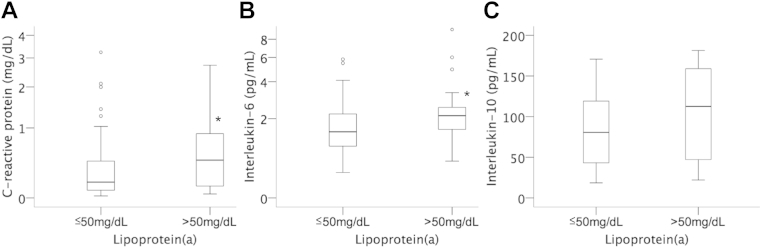
Circulating inflammatory markers in patients with normal and elevated lipoprotein(a). Plasma levels of C-reactive protein (log-scale) (A), interleukin (IL)-6 (log-scale) (B), and IL-10 (C) were determined as described under Methods in patients with normal (≤50 mg/dL) and elevated lipoprotein(a) (>50 mg/dL). Box plots represent median, interquartile, and total range. *P < .05.

Plasma levels of the acute phase marker CRP were significantly higher in patients with elevated Lp(a) (0.46; IQR, 0.12–0.90 mg/dL) as compared with patients with low Lp(a) (0.17; IQR, 0.08–0.46 mg/dL; *P* = .042, Fig. 4). Patients with Lp(a) >50 mg/dL also showed elevated plasma levels of the proinflammatory cytokine IL-6 (2.1; IQR, 1.4–2.8 pg/mL) as compared with patients with Lp(a) ≤50 mg/dL (1.5; IQR, 1.0–2.2 pg/mL; *P* = .045). In contrast, the anti-inflammatory cytokine IL-10 was not different in patients with high Lp(a) (103.0 ± 58.7 pg/mL) as compared with patients with low Lp(a) (84.5 ± 45.3 pg/mL; *P* = .23). Stepwise multivariate linear regression analysis revealed that the association between Lp(a) and IM was independent of high-sensitive CRP or IL-6, respectively.

## Discussion

In the present study, we provide evidence for the first time that in patients with stable CAD and elevated serum levels of the highly proatherogenic Lp(a), monocyte subset distribution exhibits a more proatherogenic pattern evidenced by elevated levels of the proinflammatory subset of IM, defined as CD14++CD16+ cells that has been shown to be predictive for cardiovascular events.^19^ This difference was independent of clinical risk factors such as gender, hypertension, smoking status, and levels of TC and was not influenced by the choice of statin treatment regime. In addition, we were able to demonstrate that proinflammatory OxPLs are increased in patients with elevated Lp(a) and that OxPL/apoB correlated with IM.

Already more than 2 decades ago, heterogeneity among monocytes was first described by Passlick et al^9^ using the surface expression pattern of CD14 and CD16. The newly described CD16-positive cells received widespread attention and were soon described as potent proinflammatory cells as the proportion of these cells was increased in many diseases with an underlying acute or chronic inflammatory stimulus, including atherosclerotic disease.^11,12^ Furthermore, these cells exhibit a stronger inflammatory answer after activation with various stimuli.^10^ About a decade later, by analyzing chemokine receptor expression on CD16-positive monocytes, Ancuta et al provided evidence for a “subset in the subset,” thus laying the ground for the distinction between CD14++CD16+ and CD14+CD16++ monocytes, which was finally officially acknowledged in 2010.^13,28^ The newly termed “IMs” feature a strong inflammatory potential as they strongly produce reactive oxygen species, IL-1β, and tumor necrosis factor-α.^29,30^ In a study including more than 900 stable CAD patients undergoing elective coronary angiography, IMs independently predicted cardiovascular events.^19^

The plasma lipoprotein Lp(a) has been extensively described as an independent risk factor for both the development of stable atherosclerotic disease and the sudden onset of acute ischemic events including myocardial infarction.^2,4,31^ The underlying pathophysiological mechanisms for the enhanced atherothrombotic potential of Lp(a) include a facilitated entrapment within the intima, promotion of monocyte adhesion to the endothelial monolayer, an enhanced binding capacity of OxPLs, and antifibrinolytic and prothrombotic effects.^25,32–37^ Still, consensus among the respective cardiovascular societies on when and in whom to measure Lp(a) and how to react to elevated levels is missing. The aforementioned European Atherosclerosis Society Consensus Panel agreed on measuring Lp(a) once in individuals at high or intermediate risk for CVD including familial hypercholesterolemia and in patients with a positive family history of premature atherosclerotic disease and a 10-y risk of fatal CVD of 3% or more.^2^ They further recommend a desirable level for Lp(a) below the 80th percentile, corresponding to less than 50 mg/dL.

Various cross-sectional and interventional studies in different patient cohorts demonstrated monocyte subset-specific interactions with different lipoprotein subclasses. When comparing findings from today to earlier studies, it has to be noted that many previous studies only discriminated between CD14-positive and CD16-positive cells as evidence for a further division of CD16-positive cells was only acknowledged recently.^13^ For instance in a smaller cross-sectional study, an inverse correlation between NCM and HDL was shown, whereas a follow-up study of the same group could not confirm this observation.^17,18^ However, in the latter study, positive correlations between NCM and TC, LDL cholesterol, and triglycerides were observed.^18^

In our study, when patients were stratified according to their serum Lp(a) levels above or below the threshold of 50 mg/dL into 2 groups, patients with Lp(a) serum levels above 50 mg/dL, which corresponds to the 80th percentile calculated from the Copenhagen General Population Study,^38^ demonstrated a striking shift toward a more inflammatory monocyte subset distribution. In brief, the proportion of IMs was strongly increased by approximately 35% from 5.2% to 7.0% of all monocytes (*P* = .026). In contrast, CMs remained unchanged (82% in both groups), whereas the fraction of NCMs showed a trend toward a decrease. As evidenced by multivariate regression analysis, these associations were independent from clinical risk factors such as gender, age, smoking status, and LDL cholesterol levels. Furthermore, the choice of statin treatment regime did not influence the association between Lp(a) and IMs. This finding is potentially of great interest, as various studies examining a possible effect of statin treatment on monocyte subset distribution provided conflicting results.^14,18,39,40^

OxPL are proatherogenic by activating proinflammatory genes, leading to activation of inflammatory cascades within the arterial wall.^20^ Recently, it has been shown that OxPL changes the phenotype of macrophages in vitro.^41^ To examine by which mechanism Lp(a) could shift monocyte subsets toward the proatherogenic distribution, we measured OxPL bound to apolipoprotein B-100. In line with previous studies^20^ that demonstrated that Lp(a) is a major carrier of OxPL/apoB, we could show that patients with elevated Lp(a) show increased OxPL/apoB levels. In addition, OxPL/apoB was associated with IM but not with CM and NCM suggesting that the shift to a more proinflammatory monocyte subset distribution in patients with elevated Lp(a) could possibly be because of its OxPL content.

Current consensus suggests that in early stages of atherogenesis, inflammatory activation of the vessel wall attracts monocytes toward the endothelium causing migration of monocytes into the subendothelium. Once resident, they quickly evolve to macrophages starting to take up (oxidized) phospholipids. Interestingly, macrophages also exhibit heterogeneity and plasticity, the most widely recognized subsets are the M1 and M2 phenotype. The classically activated macrophage subset M1 is characterized by increased secretion of proinflammatory cytokines, reactive oxygen species, and tissue degenerating proteins such as metalloproteinases and was associated with plaque destabilization and rupture. M2 macrophages on the other hand are characterized by rather anti-inflammatory and tissue regenerating effects.^8,42^ There is currently insufficient data in the literature whether certain monocyte subsets evolve into distinct macrophage phenotypes once resident in the tissue or whether this is characterized by high plasticity. Therefore, one could only speculate whether the observed association between IM and Lp(a) has any effect on macrophage subtype distribution within the atherosclerotic plaque, let alone any functional consequences. Furthermore, only limited data are available on the possible impact of lipids on macrophage phenotype.^43^ Interestingly, in a murine experiment, it was shown that OxPLs skew macrophages to a phenotype distinct from previously described phenotypes, termed the Mox phenotype.^41^ We therefore refrain from drawing any functional consequences from our observed associations to macrophage subtype distribution within the atherosclerotic plaque.

We further analyzed whether patients with elevated Lp(a) levels demonstrate evidence of elevated chronic inflammation beyond monocyte subset distribution. Indeed, we found that these patients have elevated levels of CRP and IL-6, whereas the anti-inflammatory cytokine IL-10 did not differ from patients with Lp(a) serum levels below the threshold. A relation of Lp(a) particle levels with underlying inflammatory activation has been proposed in some patient cohorts, whereas other studies resulted in conflicting results.^44,45^ In a general Caucasian population including approximately 35,000 individuals, only a very moderate association between Lp(a) levels and levels of CRP could be found.^46^ Interestingly, linear regression analysis revealed that the association between circulating IM and Lp(a) was independent of CRP and IL-6 suggesting a possible direct effect of Lp(a) on monocyte subset distribution. It is of interest that in a recently published study, IL-6 receptor expression on CM and IM was increased in CAD patients when compared with healthy individuals.^47^

Limitations of the present study include the rather small number of patients and the cross-sectional study design that only allows us to describe associations between circulating immune cells and lipoprotein subfractions, whereas functional insights into the mechanisms underlying this observation cannot be made. Still, our results provide interesting insights into the relationship between innate immune protagonists and a dysregulated lipid status.

To conclude, our results provide evidence for an association between elevated levels of the atherogenic Lp(a) and an increase of proinflammatory monocytes of the intermediate subtype in patients with stable atherosclerotic disease. These findings suggest a potential link between elevated Lp(a) and activity of innate immunity. Further studies are needed to verify these results and elucidate the mechanisms underlying these associations.

## Figures and Tables

**Table 1:** Clinical characteristics of the population studied

Parameter	Total (n = 90)	Lp(a) ≤50 mg/dL (n = 69)	Lp(a) >50 mg/dL (n = 21)	*P* value
Lp(a) (mg/dL)	14.4 (8.3–48.5)	10.3 (6.6–18.8)	77.3 (61.0–95.2)	—
Age (y)	64.1 ± 9.0	65.1 ± 9.1	61 ± 9.7	.08
Male gender, n (%)	72 (80)	56 (81.2)	16 (76.2)	.62
Hypertension, n (%)	80 (89)	59 (85.5)	21 (100)	.06
Diabetes mellitus, n (%)	27 (30)	21 (30.4)	6 (28.6)	.87
Current smoker, n (%)	21 (23.3)	15 (21.7)	6 (28.6)	.52
CAD extent (VD)				.34
1 VD, n (%)	25 (28)	18 (26.1)	7 (33.3)	
2 VD, n (%)	36 (40)	26 (37.7)	10 (47.6)	
3 VD, n (%)	29 (32)	25 (36.2)	4 (19)	
Statin treatment				.87
No statin, n (%)	15 (17)	12 (17.4)	3 (14.3)	
Low-dose statin, n (%)	47 (52)	35 (50.7)	12 (57.1)	
Moderate-to-high dose statin, n (%)	28 (31)	22 (31.9)	6 (28.6)	
BMI (kg/m^2^)	29 ± 4.7	28.9 ± 4.4	29.5 ± 5.6	.65
HbA1c (%)	6.1 ± 0.9	6.0 ± 0.8	6.4 ± 1.2	.89
Creatinine (mg/dL)	1.1 ± 0.3	1.1 ± 0.3	1.0 ± 0.2	.16
Leukocytes (g/L)	7.1 ± 1.7	7.1 ± 1.8	7.0 ± 1.6	.93
Triglycerides (mg/dL)	130 (103–176)	131 (101–172)	126 (108–180)	.92
Total cholesterol (mg/dL)	164.6 ± 39	163.2 ± 38.4	169.2 ± 41.8	.54
HDL (mg/dL)	40.1 ± 13.4	40.6 ± 13.2	41.6 ± 14.4	.77
VLDL (mg/dL)	28.6 ± 9.4	28.4 ± 10.0	29.1 ± 7.2	.75
Non-HDL (mg/dL)	123.7 ± 36.4	122.5 ± 36.3	127.6 ± 37.3	.58
LDL (mg/dL)	93.3 ± 30.8	91.7 ± 29.1	98.2 ± 36.2	.4

BMI, body mass index; CAD, coronary artery disease; HbA1c, glycated hemoglobin; HDL, high-density lipoprotein; LDL, low-density lipoprotein; Lp(a), lipoprotein(a); VD, vessel disease; VLDL, very low–density lipoprotein. Values are given in mean ± standard deviation for parametric or median (interquartile range) for nonparametric data. Statin dose: Moderate-to-high dose statin treatment was defined as treatment with atorvastatin with a dosage of at least 40 mg or rosuvastatin at a dosage of at least 10 mg daily.

**Table 2:** Multivariate linear regression model for the association of lipoprotein(a) and circulating intermediate monocytes

Parameter	Univariate *P* value	β	*P* value
Lp(a) >50 mg/dL	.014	0.22	.044
Smoking	.024	0.22	.029
LDL cholesterol	.053	0.18	.13
Gender	.12	0.11	.28
Age	.98	0.10	.39
Statin dose	.45	−0.14	.43
Total model *R*^2^		0.17	.018

LDL, low-density lipoprotein. Statin dose: high-dose statin treatment was defined as treatment with atorvastatin with a dosage of at least 40 mg or rosuvastatin at a dosage of at least 10 mg daily.
